# A Diagnostic Formula for Discrimination of Tuberculous and Bacterial Meningitis Using Clinical and Laboratory Features

**DOI:** 10.3389/fcimb.2019.00448

**Published:** 2020-01-17

**Authors:** Yun Yang, Xin-Hui Qu, Kun-Nan Zhang, Xiao-Mu Wu, Xin-Rong Wang, An Wen, Ling-Juan Li

**Affiliations:** ^1^Department of Neurology, Jiangxi Provincial People's Hospital Affiliated to Nanchang University, Nanchang, China; ^2^Department of Prenatal Diagnosis, Jiangxi Maternal and Child Health Hospital, Nanchang, China

**Keywords:** tuberculous meningitis, bacterial meningitis, diagnosis, clinical features, laboratory features

## Abstract

**Background:** The discrimination of tuberculous meningitis and bacterial meningitis remains difficult at present, even with the introduction of advanced diagnostic tools. This study aims to differentiate these two kinds of meningitis by using the rule of clinical and laboratory features.

**Methods:** A prospective observational study was conducted to collect the clinical and laboratory parameters of patients with tuberculous meningitis or bacterial meningitis. Logistic regression was used to define the diagnostic formula for the discrimination of tuberculous meningitis and bacterial meningitis. A receiver operator characteristic curve was established to determine the best cutoff point for the diagnostic formula.

**Results:** Five parameters (duration of illness, coughing for two or more weeks, meningeal signs, blood sodium, and percentage of neutrophils in cerebrospinal fluid) were predictive of tuberculous meningitis. The diagnostic formula developed from these parameters was 98% sensitive and 82% specific, while these were 95% sensitive and 91% specific when prospectively applied to another 70 patients.

**Conclusion:** The diagnostic formula developed in the present study can help physicians to differentiate tuberculous meningitis from bacterial meningitis in high-tuberculosis-incidence-areas, particularly in settings with limited microbiological and radiological resources.

## Introduction

Tuberculous meningitis (TBM) is a worldwide disease with a case fatality rate of 15–68% (Sheu et al., 2009; Christensen et al., 2011; Bahr et al., 2018; Thakur et al., 2018), and more than half of survivors are left with neurological sequelae (Hosoglu et al., 2002; Chiang et al., 2014; Thakur et al., 2018). The early diagnosis and treatment of tuberculous meningitis has long been recognized as the most important prognostic factor (Hosoglu et al., 2002; Katti, 2004; Bahr et al., 2018). The difficulty of diagnosing tuberculous meningitis lies on its similar clinical features, including headache, fever, and vomiting, when compared to other meningoencephalitides. At present, the diagnosis of tuberculous meningitis relies on the isolation of *Mycobacterium tuberculosis* from the cerebrospinal fluid. Unfortunately, the laboratory diagnosis for TBM is expensive, time-consuming and not very sensitive. Microscopy is very insensitive, and culture is too slow for decision-making. Other new techniques, such as interferon gamma release assays (IGRAs) and GeneXpert MTB/RIF (a kind of nucleic acid amplification test for detecting DNA sequences specific for *M. tuberculosis* and rifampicin resistance) are not very sensitive (Thwaites et al., 2002; Pai et al., 2003; Bahr et al., 2016, 2018). Therefore, new, accurate, simple, and rapid diagnostic tests are required. Criteria developed to distinguish TBM and bacterial meningitis by clinical and laboratory features developed in Vietnam has been tested in different populations (Table 1; Thwaites et al., 2002). The Vietnam rule was originally described as 86% sensitive and 79% specific for TBM diagnosis in adults. However, subsequent studies in Turkey (Sunbul et al., 2005), Vietnam (Török et al., 2007), and India (Vibha et al., 2012) reported sensitivities that ranged within 96–98% and specificities that ranged within 68–88%. The major limitation of the rule was its specificity, which still needs to be elevated.

**Table 1:** The Vietnam rule for the diagnosis of TBM on admission.

**Variable**	**Score**
**Age (years)**	
≥36	2
<36	0
**Blood WCC (10**^**3**^**/ml)**	
≥15,000	4
<15,000	0
**History of illness (days)**	
≥6	−5
<6	0
**CSF total WCC (10**^**3**^**/ml)**	
≥750	3
<750	0
**CSF % neutrophils**	
≥90	4
<90	0

Suggested rule for diagnosis: total score ≤ 4 = TBM; total score > 4 = non-TBM.

*TBM, tuberculous meningitis; WCC, white cell count; CSF, cerebrospinal fluid*.

As it is known, TBM can cause many metabolic disorders, and the commonest of which is hyponatraemia, which affects more than 50% of patients with the disease (Davis et al., 1993; Misra et al., 2016; Mai and Thwaites, 2017). However, the Vietnam rule does not contain the clinical feature of hyponatraemia. Hence, the present study attempted to determine whether the clinical feature of hyponatraemia can increase the specificity of the Vietnam rule. If this would be possible, the investigators would attempt to derive a new formula for diagnosing adult TBM through the use of clinical and laboratory features.

## Methods

### Patient Recruitment

This prospective observational study was conducted in Jiangxi Provincial People's Hospital, Nanchang, Jiangxi, China. All consecutive patients admitted in the Neurology Ward of our hospital with a suspected diagnosis of central-nervous-system infection between July 2012 and September 2016 were recruited into the present study. The enrolled patients presented with at least one of the following symptoms: fever, headache, neurological deficits, or disturbance of consciousness. Based on the baseline investigations, these patients were divided into two groups according to the criteria used by Thwaites et al. (2002). Since this was a validation study, the criteria for classifying patients was the same as in the original study by Thwaites et al. (2002). The patients were classified as tuberculous meningitis when: (1) *M. tuberculosis* was isolated from the cerebrospinal fluid (that is, acid-fast bacilli was observed in the CSF, the *M. tuberculosis* is cultured from the CSF, or the CSF commercial nucleic acid amplification test was positive), or (2) clinical meningitis with negative gram and India ink stains, plus sterile bacterial and fungal cultures, and one or more of the following: The cranial magnetic resonance imaging was consistent with TBM (hydrocephalus, edema, basal meningeal enhancement, tuberculoma, and infarction), the chest radiograph was consistent with active pulmonary tuberculosis or there was clinical evidence of other extrapulmonary tuberculosis, and there was a good response to the anti-tuberculosis chemotherapy. Bacterial meningitis was diagnosed when: (1) pathogenic bacteria was isolated from the cerebrospinal fluid, or (2) clinical meningitis, with all of the following: The clinical meningitis presented with all of the following: lymphocytes and neutrophils in the cerebrospinal fluid, low concentration of glucose in the cerebrospinal fluid (<50% of that in blood), sterile blood and cerebrospinal fluid cultures, and full recovery without anti-tuberculosis chemotherapy at 3 months after admission.

### Inclusion Criteria

Patients with clinical and CSF features suggestive of community acquired meningitis of ≥12 years old were included (patients of younger age were admitted to the Pediatrics Department as a hospital policy).

### Exclusion Criteria

Any of the following: (1) Meningitis in post-operative neurosurgical condition, or post-traumatic meningitis or parameningeal infections, or brain abscess; (2) chronic meningitis other than tuberculous meningitis; (3) patients with a positive HIV result.

### Procedures

All prospectively enrolled patients underwent standard history taking and physical examination. Then, lumbar punctures were carried out, and serum antibodies to HIV, chest radiography, and magnetic resonance imaging of the head were performed. Afterwards, 12 mL of cerebrospinal fluid was centrifuged, and the deposit of 6 mL of cerebrospinal fluid was examined by microscopy with gram, Ziehl-Neelsen and India ink stains. The remaining part of deposit was cultured on blood and chocolate agar, and Lowenstein-Jensen media. The diagnostic criteria for tuberculous and bacterial meningitis were applied for all patients included in the present study.

Patients with proven or suspected bacterial meningitis were treated with 21 days of intravenous ceftriaxone (Roche, Basel, Switzerland; 3 g per day) or other intravenous antibiotics, according to the antimicrobial susceptibility results. Patients with proven or suspected TBM were treated with four drugs (isoniazid, rifampicin, streptomycin, and pyrazinamide) for 3 months, followed by three drugs (isoniazid, rifampicin, and pyrazinamide) for 6 months. Then, prednisolone was used for 4 weeks, and reduced to stop over 4 weeks. The clinical symptoms and therapeutic effects were recorded in detail.

### Statistical Analysis

The 25 clinical and laboratory parameters of patients with TBM and bacterial meningitis described in Table 2 were recorded at admission. The variables were investigated using visual (plots/histograms) and analytical methods (Kolmogorov–Smirnov Test) to determine whether these are normally distributed. The Mann-Whitney *U*-test or student *t*-test was used to compare continuous variables between the two groups. The qualitative data was analyzed by using chi-square/Fischer's exact test, wherever applicable. The odds ratio and 95% confidence interval were calculated.

**Table 2:** Univariate analysis comparing variables between patients with tuberculous and bacterial meningitis.

**Characteristic**	**Tuberculous meningitis**		**Bacterial meningitis**		***P*-value**
		***n***		***n***	
Age, years; median (90% range)	52 (19–75)	58	42 (16–71)	45	0.107
Sex, male (%)	36 (62)	58	33 (73)	45	0.228
Duration of illness, days; median (90% range)	15 (4–120)	58	3 (1–26)	45	0.0001
Duration of headache, days; median (90% range)	7 (0–50)	58	2 (0–26)	45	0.178
Duration of fever, days; median (90% range)	10 (0–63)	58	2 (0–14)	45	0.0001
Coma before admission (%)	17 (29)	58	14 (31)	45	0.843
Hemiplegia (%)	8 (14)	58	4 (9)	45	0.442
Cranial nerve palsies (%)	7 (12)	58	7 (16)	45	0.609
Recent loss of weight (%)	8 (14)	58	1 (2)	45	0.087
Night sweats (%)	6 (10)	58	0 (0)	45	0.072
Coughing for 2 or more weeks (%)	15 (26)	58	1 (2)	45	0.001
GCS; median (90% range)	15 (9–15)	58	15 (6–15)	45	0.195
Meningeal signs (%)	42 (72)	58	43 (96)	45	0.002
HCT %; median (90% range)	35 (26–43)	58	39 (22–49)	45	0.005
Blood WCC (10^3^/mL); median (90% range)	7,700 (3,385–15,735)	58	13,000 (5,300–30,480)	45	0.0001
Blood % neutrophils; median (90% range)	81 (50–95)	58	89 (71–97)	45	0.0001
Blood sodium, mmol/L; median (90% range)	133 (120–142)	58	138 (134–143)	45	0.0001
CSF opening pressure, cm H20; median (90% range)	18 (8–40)	56	20 (9–34)	45	0.559
Clear CSF appearance (%)	51 (91)	56	26 (58)	45	0.0001
CSF total WCC (10^3^/mL); median (90% range)	40 (2–448)	56	350 (7–4,852)	45	0.0001
CSF % neutrophils; median (90% range)	34 (0–75)	54	80 (15–94)	45	0.0001
CSF % lymphocytes; median (90% range)	60 (18–95)	54	20 (4–82)	45	0.0001
CSF protein, g/dL; median (90% range)	1,274 (214–2,810)	56	1,350 (584–3,388)	45	0.163
CSF/blood glucose; median (90% range)	0.3 (0.12–0.7)	56	0.25 (0.02–0.9)	45	0.129
CSF chloride, mmol/L; median (90% range)	115 (98–130)	56	118 (107–129)	45	0.023

The logistic regression (forward) was used in the univariate and multivariate analysis. The logistic regression equation of the joint probability of valuable variables was obtained to predict the probability of having TBM. A receiver operator characteristic (ROC) curve was established on the joint probability of valuable variables to determine the best cutoff point.

On the other hand, classification trees (classification and regression tree, CART) were used to divide the two groups (TBM group and bacterial meningitis group) according to the best separation for each variable. Then, continuous variables were converted to categorical variables on the basis of the best separation for each variable, and *X*^2^-test (or Fisher's exact test for small proportions) was used to compare the categorical variables. The logistic regression was used again to construct the model of the probability of having TBM. The rounded β-coefficients in the model were used to define the diagnostic index (DI) for the categorical variables. The total diagnostic index (TDI) was determined by adding up each diagnostic index for each patient, and was defined as a formula for diagnosing adult TBM. A ROC curve was established on the total diagnostic indices to determine the best cutoff point.

Finally, the sensitivity, specificity, positive predictive values (PPV), and negative predictive values (NPV) of these two methods were determined. The diagnostic formula was reevaluated by enrolling another 70 patients using the same method. All analyses were performed using SPSS 19. *P* < 0.05 was considered statistically significant.

## Results

**Figure 1:**
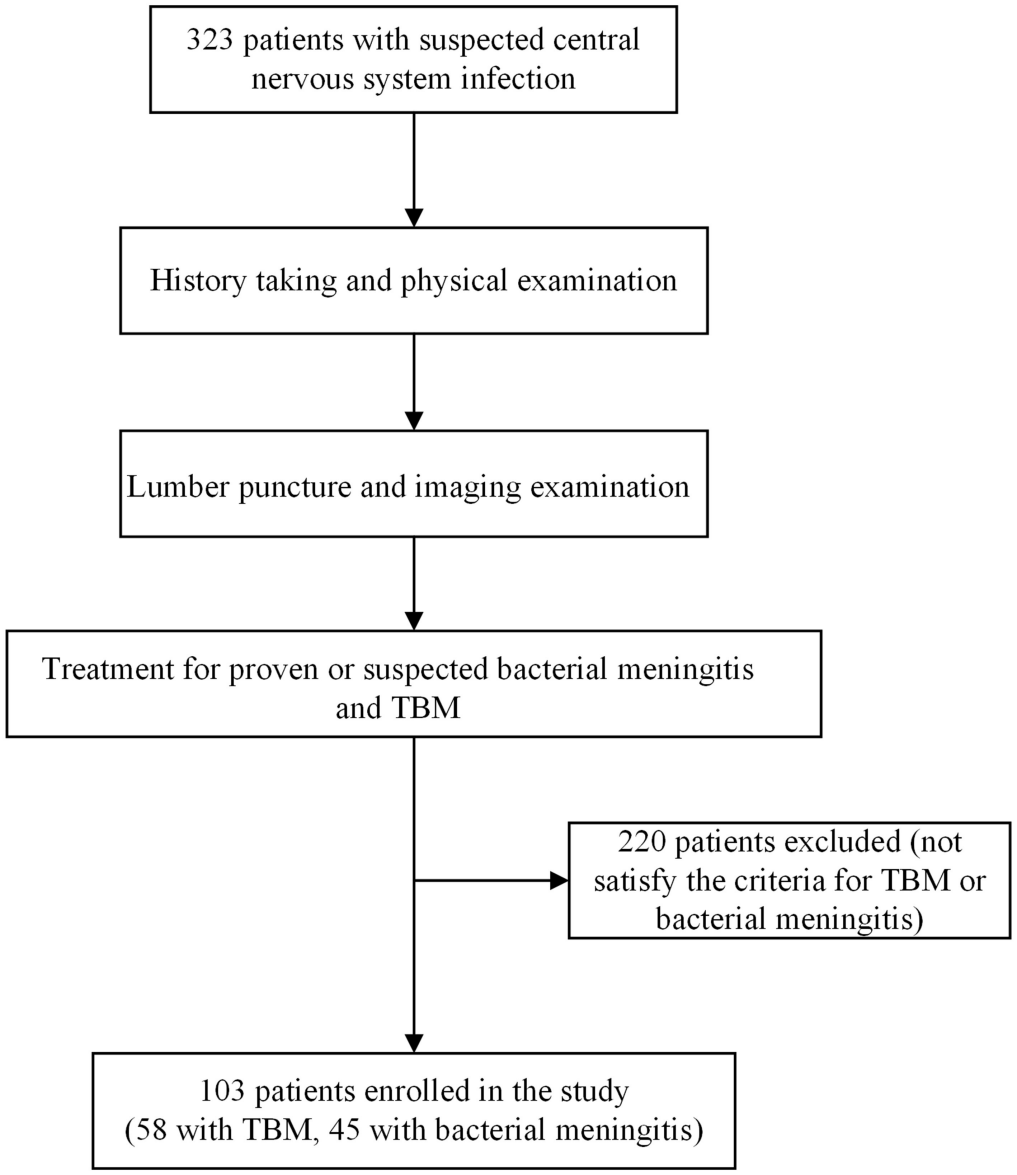
Study flowchart.

A total of 323 patients were enrolled in the present study. Among these patients, 58 patients were diagnosed with TBM, while 45 patients were diagnosed with bacterial meningitis (Figure 1). The remaining 220 patients were excluded from the present study, because they did not satisfy the above criteria for TBM or bacterial meningitis. The antibodies to HIV-1 in all the patients were negative. All patients enrolled in the present study had wet cough, but none of these patients had the symptom of haemoptysis.

Six patients were diagnosed as confirmed TBM on the basis of *M. tuberculosis* isolated from the cerebrospinal fluid, while 52 patients were diagnosed with clinical TBM. Furthermore, cranial radiological features consistent with TBM were present in 36 of 58 patients with TBM. In addition, chest radiological features consistent with active pulmonary tuberculosis were present in 13 of 58 patients with TBM. However, no evidence of extrapulmonary tuberculosis was present. Furthermore, no significant differences were found in all parameters between the confirmed TBM and clinical TBM.

A total of 20 patients were diagnosed with confirmed bacterial meningitis on the basis of the pathogenic bacteria isolated from the cerebrospinal fluid. Among these patients, 10 patients had *Staphylococcus aureus*, six patients had *Streptococcus pneumoniae*, and four patients had *Escherichia coli*. Furthermore, 25 patients, including those who were partly treated, were diagnosed with clinical bacterial meningitis. However, no significant differences were found in all these parameters between confirmed bacterial meningitis and clinical bacterial meningitis.

Among these TBM patients, three patients had third nerve palsy, two patients had sixth nerve palsy, and two patients had seventh nerve palsy. In the bacterial meningitis group, three patients had third nerve palsy, one patient had sixth nerve palsy, and three patients had seventh nerve palsy.

The clinical and laboratory parameters of TBM and bacterial meningitis are presented in Table 2. Significant differences were found in the following parameters: duration of illness, duration of fever, coughing for 2 or more weeks, meningeal signs, packed-cell volume, white blood cell count, percentage of neutrophils, blood sodium, clear cerebrospinal fluid appearance, cerebrospinal fluid total white-cell count, percentage of neutrophils in the cerebrospinal fluid, percentage of lymphocytes in the cerebrospinal fluid, and cerebrospinal fluid chloride.

A multivariate analysis was performed to derive the formula. Five variables (duration of illness, coughing for 2 or more weeks, meningeal signs, blood sodium, and percentage of neutrophils in cerebrospinal fluid) were found to be independently associated with TBM according to the stepwise forward logistic regression analysis (Table 3). Then, the logistic regression equation for determining the joint probability of the five above variables (*P*) was obtained to predict the probability of having TBM:

**Table 3:** Multivariate logistic regression analysis of original data.

	**β-coefficient**	**Odds ratio (95% CI)**	***P*-value**
Duration of illness	0.190	1.209 (1.064, 1.374)	0.004
Coughing for 2 or more weeks	4.278	72.071 (1.876, 2,768.705)	0.022
Meningeal signs	−3.034	0.048 (0.003, 0.802)	0.035
Blood sodium	−0.383	0.682 (0.539, 0.862)	0.001
CSF % neutrophils	−0.065	0.937 (0.900, 0.975)	0.001

*Values of five variables found to be associated independently with tuberculous meningitis on logistic regression analysis*.

In (P/1−P) = Logit(P) = 55.801 + 0.19 (duration for illness) + 4.278 (coughing for two or more weeks) − 3.034 (meningeal signs) − 0.383 (blood sodium) − 0.065 (CSF% of neutrophils).

**Figure 2:**
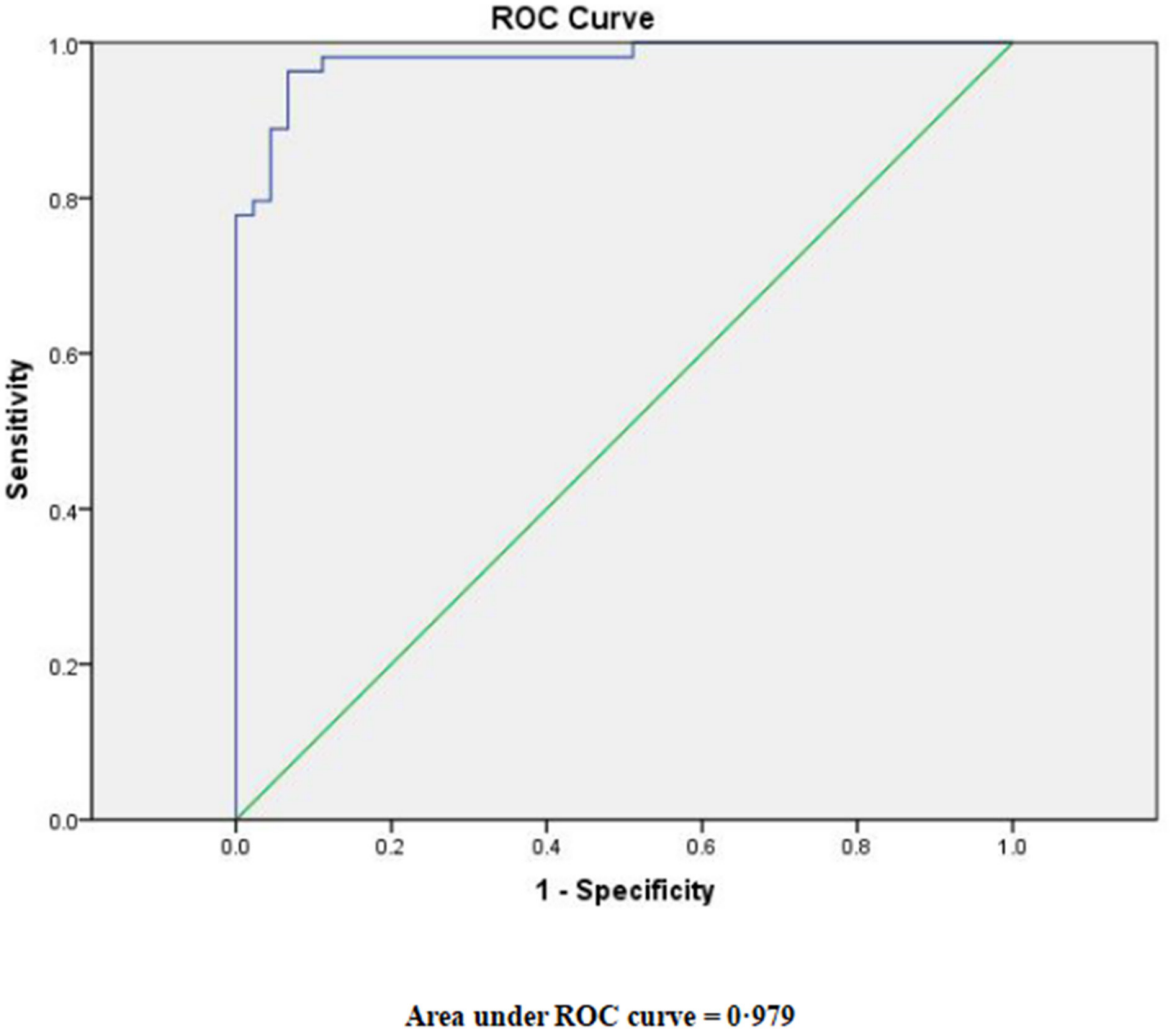
Receiver-operator characteristic (ROC) curve for the joint probability derived from the logistic regression model.

The best cutoff point for the joint probability was found to be 0.4528395, according to a ROC curve (Figure 2). TBM was considered when the joint probability was more than 0.4528395, while bacterial meningitis was considered when the joint probability was <0.4528395. Furthermore, the diagnostic sensitivity was 0.963, while the diagnostic specificity was 0.933.

**Figure 3:**
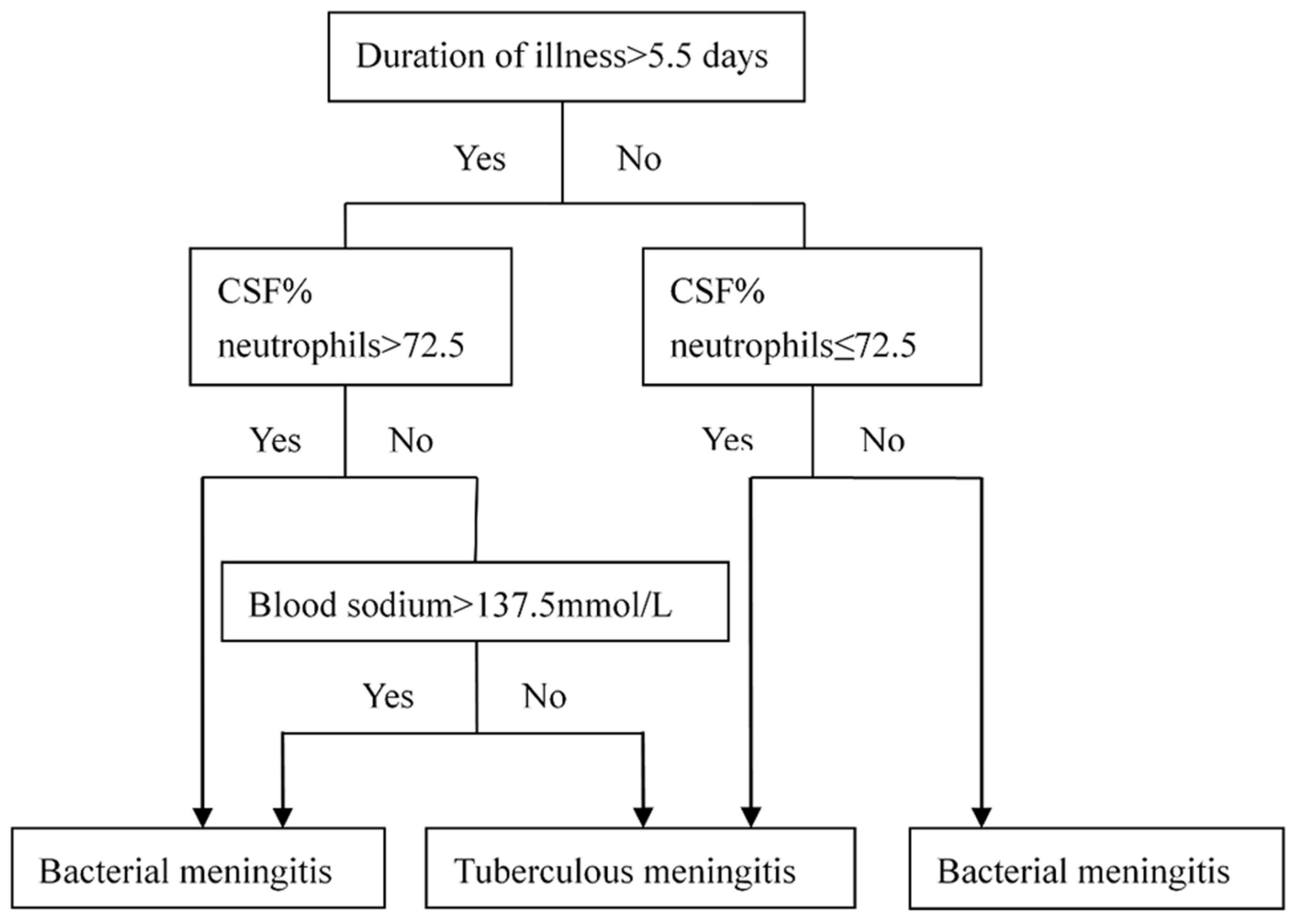
Diagnostic classification trees for use.

Next, classification trees were used to distinguish the TBM group from the bacterial meningitis group (Figure 3). According to the best separation generated from the classification trees, continuous variables, such as duration of illness, the percentage of neutrophils in the cerebrospinal fluid and blood sodium, were converted to categorical variables. The logistic regression analysis was used again to establish the model for the probability of having TBM after the variables were converted into categorical variables (Table 4). Four variables (duration of illness for more than 5.5 days, coughing for two or more weeks, blood sodium for more than 137.5 mmol/L, and the percentage of neutrophils in the cerebrospinal fluid for more than 72.5%) were found to be independently associated with TBM. The diagnostic index (DI) for the four variables are presented in Table 5.

**Table 4:** Multivariate logistic regression analysis of the converted categorical variables.

	**β-coefficient**	**Odds ratio (95% CI)**	***P*-value**
Duration of illness for more than 5.5 days	4.367	78.836 (7.387, 841.329)	0.001
Coughing for 2 or more weeks	3.860	47.481 (0.643, 3,505.548)	0.048
Blood sodium for more than 137.5 mmol/L	−3.673	0.025 (0.002, 0.289)	0.003
CSF % neutrophils for more than 72.5%	−5.213	0.005 (0.000, 0.095)	0.001

*Values of four variables found to be associated independently with tuberculous meningitis on logistic regression analysis*.

**Table 5:** Weighted diagnostic index scores for clinical variables used for diagnostic rule.

**Clinical variables**	**Diagnostic index**
**Duration of illness (days)**	
>5.5	4
≤5.5	0
**Duration of coughing (weeks)**	
≥2	4
<2	0
**Blood sodium (mmol/L)**	
>137.5	−4
≤137.5	0
**CSF % neutrophils**	
>72.5	−5
≤72.5	0

Total diagnostic index (TDI) = DI (duration of illness for more than 5.5 days) + DI (coughing for two or more weeks) + DI (blood sodium for more than 137.5 mmol/L) + DI (percentage of neutrophils in the cerebrospinal fluid for more than 72.5%).

**Figure 4:**
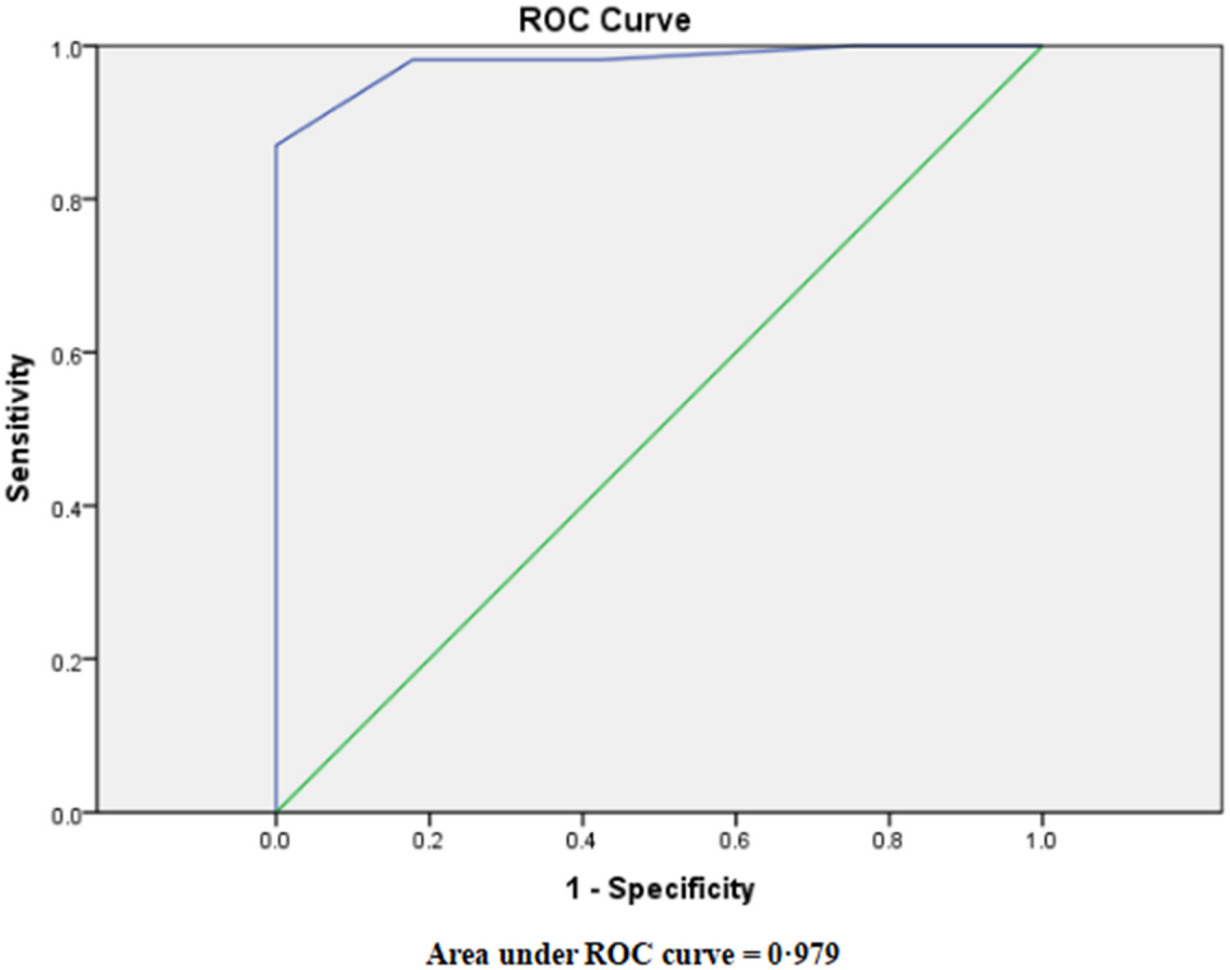
Receiver-operator characteristic (ROC) curve for the prognostic index derived from the logistic regression model.

The best cutoff point for the TDI was indicated to be 0 on the basis of the ROC curve (Figure 4). TBM was considered when TDI was 0 or more, while bacterial meningitis was considered when TDI was <0. The diagnostic sensitivity was 0.98, and the diagnostic specificity was 0.82. The PPV was 0.88 and the NPV was 0.97.

At the same time, the Vietnam rule was used to test its diagnostic value in the present population cohort. It was found that in 58 patients with TBM, 58 patients were diagnosed as TBM when using the Vietnam rule. In addition, among the 45 patients with bacterial meningitis, 29 patients were diagnosed as TBM when using the Vietnam rule. In the present cohort, the diagnostic sensitivity of the Vietnam rule was 1.0, and the diagnostic specificity was 0.36. The PPV was 0.67 and the NPV was 1.00.

The Marais criteria described in Table 6 (Marais et al., 2010) was also used to re-calculate the clinical data. It was found that among the 58 patients with TBM, 56 patients were diagnosed as TBM when using the Marais criteria. Among the 45 patients with bacterial meningitis, 20 patients were diagnosed as TBM when using the Marais criteria. In the present cohort, the diagnostic sensitivity of the Marais criteria was 0.97, and the diagnostic specificity was 0.56. The PPV was 0.74 and the NPV was 0.93.

**Table 6:** The Marais criteria for the diagnosis of TBM on admission.

	**Diagnostic score**
**Clinical criteria**	**(Maximum category score** **=** **6)**
Symptom duration of more than 5 days	4
Systemic symptoms suggestive of tuberculosis (one or more of the following): weight loss (or poor weight gain in children), night sweats, or persistent cough for more than 2 weeks	2
History of recent (within past year) close contact with an individual with pulmonary tuberculosis or a positive TST or IGRA (only in children <10 years of age)	2
Focal neurological deficit (excluding cranial palsies)	1
Cranial nerve palsy	1
Altered consciousness	1
**CSF criteria**	**(Maximum category score** **=** **4)**
Clear appearance	1
Cells: 10–500 per μl	1
Lymphocytic predominance (>50%)	1
Protein concentration greater than 1 g/L	1
CSF to plasma glucose ratio of less than 50% or an absolute CSF glucose concentration less than 2.2 mmol/L	1
**Cerebral imaging criteria**	**(Maximum category score** **=** **6)**
Hydrocephalus	1
Basal meningeal enhancement	2
Tuberculoma	2
Infarct	1
Pre-contrast basal hyperdensity	2
**Evidence of tuberculosis elsewhere**	**(Maximum category score** **=** **4)**
Chest radiograph suggestive of active tuberculosis signs of tuberculosis = 2; miliary tuberculosis = 4	2/4
CT/MRI/Ultrasound evidence for tuberculosis outside the CNS	2
AFB identified or *Mycobacterium tuberculosis* cultured from another source-i.e., sputum, lymph node, gastric washing, urine, blood culture	4
Positive commercial *M. tuberculosis* NAAT from extra-neural specimen	4
**Exclusion of alternative diagnoses**	

*TST, tuberculin skin test; IGRA, interferon-gamma release assay; NAAT, nucleic acid amplification test; AFB, acid-fast bacilli*.

Finally, the present diagnostic formula was tested on another 70 patients, who satisfied the study diagnostic criteria: three patients had confirmed TBM, 35 patients had clinical TBM, 15 patients had culture-confirmed bacterial meningitis, and 17 patients had clinical bacterial meningitis. The diagnostic formula was 0.97 sensitive and 0.81 specific. The PPV was 0.86 and the NPV was 0.96.

## Discussion

At present, the diagnosis of TBM depends on the clinical features, microbiologic findings, and radiological findings. *M. tuberculosis* isolated from the cerebrospinal fluid has been considered as the gold criterion for the diagnosis of TBM. However, the level of *M. tuberculosis* in the cerebrospinal fluid is excessively low. Hence, the sensitivity of microbiologic and molecular techniques are not high. Some reports (Thwaites et al., 2000, 2004; Yasuda et al., 2002) have suggested that the sensitivity of the acid-fast bacilli of *M. tuberculosis* after Ziehl-Neelsen staining was within 10–60% in meticulous microscopy and the culture of a large volume (>5 mL) of cerebrospinal fluid. Ducomble et al. (2013) reported that the sensitivity of the *M. tuberculosis* culture was within 40–60%, and the *M. tuberculosis* culture duration was 3–8 weeks (Thwaites et al., 2002). In a meta-analysis (Pai et al., 2003) of studies that examined the use of nucleic acid amplification techniques (NAATs) for the diagnosis of TBM, the investigators calculated that commercial NAATs were 56% sensitive (95% CI: 46–66) and 98% specific (95% CI: 97–99). Furthermore, several studies have examined the diagnostic use of interferon-gamma release assays on CSF for the diagnosis of TBM (Thwaites et al., 2002; Patel et al., 2010; Vidhate et al., 2011; Park et al., 2012). Their findings suggest that these results are indeterminate unless CSF volumes of 5–10 mL are tested, and that these assays are specific (70–90%), but have low sensitivity (50–70%).

Based on the low sensitivities of these diagnostic techniques, more and more studies have attempted to distinguish TBM from bacterial meningitis using clinical and laboratory features. Thwaites et al. (2002) enrolled 251 adult meningitis patients in Vietnam to develop a simple diagnostic rule through clinical and laboratory features. The parameters of age, blood white cell count, history of illness, cerebrospinal fluid total white cell count, and percentage of neutrophils in the cerebrospinal fluid were found to be independently associated with TBM. Furthermore, the Vietnam rule had a sensitivity of 97% and a specificity of 91%. After applying this to another 75 patients, the Vietnam rule had a sensitivity of 86% and a specificity of 79%. In the present population cohort, the specificity of the Vietnam rule was only 36%. It was concluded that the reason for the low specificity was that a large part of the present population cohort was treated before they were sent to our hospital. Hence, the parameters of blood white cell count and CSF total white cell count in the Vietnam rule were not accurate. Therefore, when encountering many partly treated patients, the Vietnam rule is always not very specific. Ersoy et al. (2012) retrospectively enrolled 96 adult meningitis patients in Turkey to modify the Vietnam rule. The parameter of serum C-reactive protein (CRP) level was added to the diagnostic rule. It was reported that the modified diagnostic rule had a sensitivity of 95.5% and a specificity of 100%. Some scholars in India (Vibha et al., 2012) have reported that the sensitivity and specificity of the new diagnostic rule was 95.7 and 97.6%, respectively, when the age factor was excluded. Dendane et al. (2013) found that the female gender was also an independent predictor of TBM, and established a new diagnostic rule that had a sensitivity of 87% and a specificity of 96%.

Hyponatraemia is very common in patients with TBM (Thwaites and Tran, 2005; Figaji and Fieggen, 2010), and it has been considered to be caused by leptomeningeal inflammation, hydrocephalous, increased intracranial pressure, and ventriculitis (Karandanis and Shulman, 1976). Either cerebral salt wasting (CSW), or the syndrome of inappropriate secretion of antidiuretic hormone (SIADH) was regarded as the possible pathogenesis of hyponatraemia (Van Embden et al., 1993; Zhang et al., 1999; Reed et al., 2004; Tsenova et al., 2005; Mihailidou et al., 2012). CSW has been considered as the combination of hypovolemia, dehydration, high urinary output, weight loss, and hyponatraemia, which is due to the secretion of some natriuretic protein by the brain or arteries (Tinggaard et al., 2011; Inamdar et al., 2016; Misra et al., 2016, 2018; Mai and Thwaites, 2017). This is sometimes due to the secretion of adrenomedullin, which is an endogenous peptide (Lang et al., 1997). SIADH has been regarded as the combination of hyperhydration and hyponatraemia, which is caused by the excess secretion of antidiuretic hormone through the hypothalamus (Van Embden et al., 1993; Zhang et al., 1999; Reed et al., 2004).

The parameter of blood sodium level was excluded due to the large number of missing values in the Vietnam diagnostic rule (Thwaites et al., 2002). In order to investigate the diagnostic value of the parameter of blood sodium level, the present study prospectively enrolled 103 adult patients with meningitis (58 patients with TBM and 45 patients with bacterial meningitis). The following diagnostic rule was achieved: TDI = DI (duration of illness for more than 5.5 days) + DI (coughing for two or more weeks) + DI (blood sodium of more than 137.5 mmol/L) + DI (percentage of neutrophils in the cerebrospinal fluid for more than 72.5%). Then, a ROC curve was drawn with a cutoff of 0, because this provides the greatest sensitivity (98%) with acceptable specificity (82%). The application of the test data revealed a 95% sensitivity and 91% specificity. When applying the Marais criteria to the present population cohort, the specificity was only 0.56. It is possible that the low specificity was due to a large part of treated patients in the present population cohort. Hence, the parameters of CSF cells and CSF protein were not so accurate. In fact, it is very difficult to distinguish partly treated bacterial meningitis with tuberculous meningitis. The present diagnostic formula was achieved based on a large amount of partly treated patients. Hence, a new excellent diagnostic formula for distinguishing TBM from bacterial meningitis by using clinical and laboratory features was derived.

There were still some limitations in the present study. First, the patients enrolled in the present study were all negative for antibodies to HIV-1. Although HIV-1 does not change the clinical and laboratory features of the disease (Beardsley et al., 2018; Ellis et al., 2018), it alters the differential diagnosis in meningitic adults: opportunistic infection with unusual pathogens must be considered, in particular Cryptococcus neoformans, which can present subacutely in a similar way to tuberculous meningitis. Therefore, the diagnostic formula in the present study is not recommended in regions that have a high incidence of acquired immune deficiency syndrome (AIDS). Second, the sample size of this study had not been calculated before the study was carried out. Although both not-treated patients and partly-treated patients were enrolled in the present study, the number of patients was not significant. Therefore, further studies involving more patients are needed to validate this new diagnostic formula.

In conclusion, the diagnostic formula developed in the present study can help physicians identify patients suffering from TBM in a short period of time, and can be used in tuberculosis high-incidence areas, particularly in settings with limited microbiological and radiological resources.

## Data Availability Statement

The raw data supporting the conclusions of this article will be made available by the authors, without undue reservation, to any qualified researcher.

## Ethics Statement

The clinical symptoms and therapeutic effects were recorded in detail. The present study was conducted in accordance with the declaration of Helsinki and approved by the Hospital Research Ethical Committee. All participants provided signed informed consent.

## Author Contributions

YY and X-HQ worked on the study design and wrote the article. K-NZ, X-MW, and X-RW worked on data collection. AW and L-JL conducted data analysis. All authors read and approved the final manuscript.

### Conflict of Interest

The authors declare that the research was conducted in the absence of any commercial or financial relationships that could be construed as a potential conflict of interest.
